# Electroconvulsive therapy is associated with increased immunoreactivity of neuroplasticity markers in the hippocampus of depressed patients

**DOI:** 10.1038/s41398-023-02658-1

**Published:** 2023-11-20

**Authors:** Dore Loef, Indira Tendolkar, Philip F. P. van Eijndhoven, Jeroen J. M. Hoozemans, Mardien L. Oudega, Annemieke J. M. Rozemuller, Paul J. Lucassen, Annemiek Dols, Anke A. Dijkstra

**Affiliations:** 1 Amsterdam UMC, location VUmc, Amsterdam, Department of Psychiatry, Amsterdam Neuroscience, Amsterdam, the Netherlands; 2 GGZ inGeest Specialized Mental Health Care, Amsterdam, the Netherlands; 3 Department of Psychiatry, Radboud University Medical Center, Nijmegen, the Netherlands; 4 Donders Institute for Brain, Cognition and Behavior, Centre for Cognitive Neuroimaging, Nijmegen, the Netherlands; 5 Department of Psychiatry and Psychotherapy, University Hospital Essen, Essen, Germany; 6 Department of Pathology, Amsterdam Neuroscience, Amsterdam University Medical Centre, Amsterdam, the Netherlands; 7 Brain Plasticity Group, Swammerdam Institute for Life Sciences, University of Amsterdam, Amsterdam, the Netherlands; 8 Department of Psychiatry, UMC Utrecht Brain Center, University Utrecht, Utrecht, the Netherlands; 9 Molecular Neuroscience Group, Swammerdam Institute for Life Sciences, Center for Neuroscience, University of Amsterdam, Amsterdam, the Netherlands

**Keywords:** Neuroscience, Depression

## Abstract

Electroconvulsive therapy (ECT) is an effective therapy for depression, but its cellular effects on the human brain remain elusive. In rodents, electroconvulsive shocks increase proliferation and the expression of plasticity markers in the hippocampal dentate gyrus (DG), suggesting increased neurogenesis. Furthermore, MRI studies in depressed patients have demonstrated increases in DG volume after ECT, that were notably paralleled by a decrease in depressive mood scores. Whether ECT also triggers cellular plasticity, inflammation or possibly injury in the human hippocampus, was unknown. We here performed a first explorative, anatomical study on the human post-mortem hippocampus of a unique, well-documented cohort of bipolar or unipolar depressed patients, who had received ECT in the 5 years prior to their death. They were compared to age-matched patients with a depressive disorder who had not received ECT and to matched healthy controls. Upon histopathological examination, no indications were observed for major hippocampal cell loss, overt cytoarchitectural changes or classic neuropathology in these 3 groups, nor were obvious differences present in inflammatory markers for astrocytes or microglia. Whereas the numbers of proliferating cells expressing Ki-67 was not different, we found a significantly higher percentage of cells positive for Doublecortin, a marker commonly used for young neurons and cellular plasticity, in the subgranular zone and CA4 / hilus of the hippocampus of ECT patients. Also, the percentage of positive Stathmin 1 cells was significantly higher in the subgranular zone of ECT patients, indicating neuroplasticity. These first post-mortem observations suggest that ECT has no damaging effects but may rather have induced neuroplasticity in the DG of depressed patients.

## Introduction

Electroconvulsive therapy (ECT) is an effective treatment modality in psychiatry, that is mostly prescribed for treatment-resistant, major depressed patients, with response rates of up to 85% [1, 2]. While its exact mechanisms of action and cellular effects remain elusive, human and animal studies have suggested a possible role for changes in neuroplasticity (e.g. neurogenesis or gliogenesis) [3]. Although it is generally considered a safe treatment [4–7], some clinicians and patients may be reluctant to start ECT in view of suspected injury, possible cognitive impairment or inflammation of the brain [8–11], also since it was so far unknown whether ECT elicits any inflammation, damage and/or neuropathological changes.

Much research into possible ECT mechanisms has focused on the hippocampus. Magnetic resonance imaging (MRI) studies have shown reductions in hippocampal volume in patients with depression [12], which might be due to reductions in neurogenesis in aspects of depression [11, 13–18]. The occurrence of neurogenesis in the human hippocampus has recently been heavily debated, and both a general absence [19–21] as well as a prominent presence have been reported [22–28]. Next to anatomical studies, other approaches like C14 carbon dating [29], magnetic resonance spectroscopy [30], in vitro studies and recently also single-cell sequencing date [31], all support the existence of neurogenesis in the human brain, as discussed recently [22, 32]. Furthermore, MRI studies on psychiatric patients have recently shown increases in hippocampal volume after ECT that were modified by electrode placement and the number of ECT sessions [4, 33, 34]. Interestingly, while the increase in the volume of the entire hippocampus was not associated with clinical outcome [33, 35], high-resolution, 7 T imaging of the hippocampal subfields revealed that a selective increase in the volume of the dentate gyrus (DG) contributed most to the overall change in hippocampal volume. Notably, this volume increase in the DG was significantly correlated with a decrease in depression scores [36], highlighting a possible role for DG plasticity in the recovery of major depressive disorder (MDD) after ECT.

These DG changes have also been supported by animal data. In a rat model, electroconvulsive stimulation (ECS) induced hippocampal mossy fiber sprouting in the subgranular zone (SGZ) of the DG, and increases in ECS frequency lead to more sprouting in the DG [37], suggesting that ECS promotes DG plasticity and possibly neurogenesis in rats. Indeed, this was confirmed by significant increases shortly after ECS in the number of proliferating cells in the rat DG, based on bromodeoxyuridine (BrdU) studies [38]. Furthermore, the epileptic responses induced by ECS were associated with increases in the expression of Doublecortin (DCX), a microtubule-associated protein expressed in migrating neuroblasts, that is often used as marker for immature neurons or neurogenesis [39, 40], both in the DG of rodents [41–48] and humans [23, 27, 49, 50]. Research in rats has further shown that ECS treatment induced an immediate glial response in several brain areas, an activation that was again diminished four weeks later [51].

It remained so far unclear, however, whether ECT also induces changes in cellular plasticity in the human hippocampus. Consistent with investigations in non-human primates [52], the typical time-to-effect of antidepressant treatment and/or ECT is generally less than two months, in line with a time frame that activated stem cells would likely need to develop into new neurons and integrate in the DG circuit. Alternatively, the volume changes after ECT could possible also relate to inflammatory changes due to e.g. activation of (astro)glia [53].

Effects of ECT on hippocampal plasticity measures have so far been mainly examined in animal models, and by means of imaging volume changes only in patients, but not in the human post-mortem brain. In order to bridge this gap, we here studied a unique cohort of depressed patients who had received varying ECT treatment regimens during their lives and investigated whether changes in proliferation and DCX expression were present in the DG. Importantly, to control for the possible influence of medication or depression on proliferation and DCX, the hippocampi of ECT-treated patients were compared to the hippocampi of medicated depressed patients not treated with ECT and neurologically healthy controls. We further assessed whether there is a relation to the number of ECT sessions and/or the time interval to the last ECT session, and explored possible neuropathological and glial changes in the hippocampi of these 3 groups.

## Methods

### Subjects

Post-mortem hippocampi were selected from the Netherlands Brain Bank (NBB), from; a) 12 depressed donors who had received ECT in the 5 years prior to their death (ECT; mean age = 54.25), b) 10 age-matched depressed donors (DC; mean age = 66.90) who did not receive ECT, and c) 15 age-matched healthy control donors (HC; mean age = 68.87) without any neurological or psychiatric history. All data and material collected by the NBB are obtained on the basis of written informed consent. All procedures involving patients were approved by the ethics committees of Amsterdam University Medical Center. Detailed clinical records were requested from the NBB to gather all possible information concerning the ECT course. The donors in the ECT group had varying durations between their last ECT and their deaths, varying from 45 months of receiving their last ECT to less than one month prior to death (Table 1). Donors in the DC group had experienced at least one reported depressive episode in the 5 years prior to their death. Most subjects with available electrode placement information had received both right unilateral (RUL) and bilateral (BL) ECT sequentially. Therefore, no clear distinction was made between RUL and BL ECT.

**Table 1:** Clinical characteristics of donors who received ECT during life.

Case	Gender	Age of death	Diagnosis	ECT course(s)	Electrode placement	Total number of ECT sessions	Time between last ECT death in months	Remission to ECT
1	Male	55	Bipolar disorder	• Age 51: 7 ECT sessions • Age 53: 30 sessions maintenance ECT (1x/month)	• Unknown • Unknown	37 sessions	29 months	Unknown
2	Male	73	Bipolar disorder and dementia	• Age 43: treatment with ECT • Age 68: 12 sessions ECT • Age 70: 7 sessions ECT	• Unknown • RUL • Unknown	19 sessions in the last 5 years before his death	34 months	Remitted
3	Female	100	MDD	• ± Age 90: start of 66 ECT sessions • Age 93-94: 29 ECT sessions (first 1x/week, followed by 1x/2weeks) • Age 94-98: 81 ECT sessions (1x/2weeks) • From age 98 till death: maintenance ECT (1x/month)	• Unknown • BL • RUL • RUL	Approximately 200 sessions	≤1 month	Unknown
4	Female	47	MDD	• Age 44: 8 ECT sessions (2x/week) • Age 44: next 22 ECT sessions (1x/week) followed by 10 ECT sessions (2x/week)	• Unknown • Unknown	40 sessions	35 months	Nonresponse
5	Male	60	MDD and autoimmune encephalitis	• Age 58-59: 16 ECT sessions	• 10 RUL + 6 BL	16 sessions	13 months	Partially remitted
6	Male	58	MDD, personality disorder	• Age 53: 10 ECT sessions • Age 53: 6 ECT sessions • Age 54: 8 ECT sessions • Age 54: 21 ECT sessions • Age 56-57: 22 ECT sessions • Age 57: followed by maintenance ECT (first 1x/week, next 1x/2weeks, finally 1x/3weeks)	• RUL • RUL • RUL • 14 RUL + 7 BL • 5 RUL + 17 BL • Unknown	Approximately 82 sessions	7 months	Partially remitted
7	Female	45	Bipolar II disorder, OCD, eating disorder	• Age 17: treatment with ECT • Age 42: 32 ECT sessions	• Unknown • RUL	32 sessions in the last 5 years before her death	25 months	Partially remitted
8	Female	46	Bipolar II disorder and personality disorder	• Age 44-45: 50 ECT sessions	• First month RUL followed by BL	50 sessions	± 17 months	Remitted
9	Female	23	MDD, eating disorder	• Age 20: treatment with ECT • Age 22: treatment with ECT	• Unknown • Unknown	Approximately 20 sessions	13 months	Unknown
10	Male	48	MDD	• Age 48: 12 ECT sessions	• RUL	12 sessions	29 days	Non-response
11	Male	75	MDD and MS	• Age 72: 2 ECT sessions	• Unknown	2 sessions	45 months	Non-response
12	Male	21	MDD and ASD	• Age 20: 20 ECT sessions	• Unknown	20 sessions	11 months	Remitted

*ECT* electroconvulsive therapy, *MDD* major depressive disorder, *OCD* obsessive-compulsive disorder, *MS* multiple sclerosis, *ASD* Autism Spectrum Disorder, *RUL* right unilateral electrode placement, *BL* bitemporal electrode placement.

Based on the information in their clinical records, donors who had received ECT were subdivided over either a remitted, a partially remitted or a nonresponse group. If no validated depression severity scale was available, the ECT donor was classified by a trained psychiatrist based on the descriptive outcome in his/her clinical records. When available, both the left and right hippocampus of the ECT donors were included and then averaged for quantitative analysis. This method was chosen to maximize the inclusion of these unique cases, as both hemispheres were not always available for all ECT donors.

Except for 1 donor whose medication status was unknown, all donors with a depressive disorder (both ECT donors and depressive control donors) received antidepressant medication and/or mood stabilizers. In the last 5 years prior to their death, serotonin-noradrenaline reuptake inhibitors and monoamine oxidase inhibitors were only taken by donors in the ECT group, whereas both the DC and ECT groups had been treated with selective serotonin reuptake inhibitors, tricyclic antidepressants, atypical antidepressants, lithium, and antipsychotics at similar stages in their disease process. Therefore, given their similarity in antidepressant medication history, these groups could be compared to examine the effects of ECT per se.

### Immunohistochemistry

For our immunocytochemical studies, we studied 8 µm thick sections of the formalin-fixed, paraffin-embedded hippocampus from donors of all 3 groups. Brains were fixed for 4 weeks in neutral buffered 10% formalin. For most donors, the mid-level of the hippocampus was studied, but in 21.6% of cases (HC: 2 out of 15; DC: 3 out of 10; ECT: 3 out of 12) this region was not available and an adjacent more anterior part was included for analysis, except for 1 ECT donor were a more posterior part was studied.

Cytoarchitectural and neuropathological changes of all hippocampi were examined in detail by an experienced neuropathologist (AR) according to the standard NBB protocol [54, 55], and any specific remarks regarding their histo/neuropathological details are listed in Supplementary Table 1. Using classic conventional histological and neuropathological staining for H&E, Nissl, Bodian Silver, Amyloid beta, phosphorylated tau (AT8)), and according to standard protocols, brain sections from all donor groups were studied for gross morphological aberrations in cytoarchitecture, such as a possible ectopic location of cells, overt malformations or region-specific cell loss, and for the presence of neurodegenerative changes, such as amyloid deposits or neurofibrillary tau.

Immunohistochemistry was performed using antibodies against DCX to visualize immature/young neurons (Mab Signaling Technologies, batch #4604, Danvers, MA, USA), against Stathmin 1 (STMN1) to visualize cells in transition from neuronal precursors to postmitotic neurons (ab52630; Abcam) [56], and against Ki-67 to visualize cell proliferation (MIB-1: DAKO, Glostrup, Denmark) in the granule cell layer (GCL) and SGZ of the DG. DCX is a microtubule-associated protein expressed in migrating neuroblasts that is frequently used as a proxy to detect neurogenesis in rodents [46–48] and that is also expressed in the human hippocampus, for which post-mortem delay and fixation can affect immunoreactive signal quality, as discussed elsewhere [23, 24, 57–59]. Notable, due to their rapid autopsy program, tissues from the NBB generally have a relatively short post-mortem delay, benefitting tissue and antigen preservation.

STMN1 is a phosphoprotein that plays a critically important role in the regulation of the microtubule cytoskeleton and the cell cycle, particularly during cell division [60]. STMN1 expression has a positive correlation with cellular proliferation, as interfering with its function by forced expression or inhibition leads to decreased cellular proliferation [60]. Furthermore, STMN1 is upregulated during neuronal differentiation and plasticity [61].

The Ki-67 antigen is a DNA-binding protein complex present in the nucleus of all proliferating cells during the G1, S, G2 and M, but not G0, phases of the cell cycle. Its deletion suppresses cell division in cell lines, indicating an important role in cell cycle control [62]. Ki-67 exhibits phase-specific staining patterns [24, 63] and is generally absent from quiescent, apoptotic or post-mitotic cells [58, 64, 65].

Furthermore, we explored whether possible inflammatory changes had been induced after ECT and used immunocytochemistry for glial fibrillary acidic protein (GFAP: clone EP672Y, Roche, Basel, Switzerland) to visualize astrocytes, and for ionized calcium binding adaptor molecule 1 (Iba1: Wako Pure Chemical Industries, Osaka, Japan) to identify microglia.

**Fig. 1:**
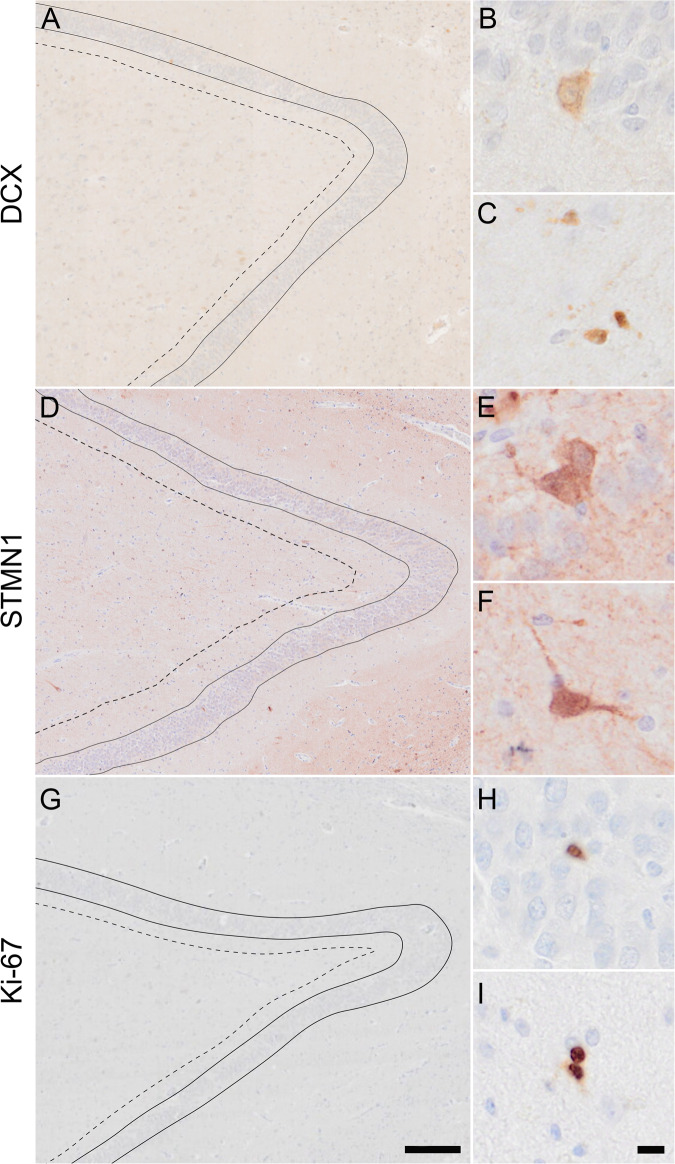
Doublecortin (DCX), Stathmin 1 (STMN1), and Ki-67 expression in the hippocampus. The granule cell layer (GCL) is delineated with a solid line and the dashed line indicates the border of the subgranular zone (SGZ; **A**, **D**, **G**). Cytoplasmic and dendritic DCX expression is shown in the GCL (**B**) and the SGZ (**C**), cytoplasmic STMN1 expression is shown in the GCL (**E**) and in the SGZ (**F**), and nuclear Ki-67 expression is shown in neurons of the GCL (**H**) and in pairs in the SGZ (**I**). Scale bar **A**, **D**, **G** = 200 µm, scale bar **B**, **C**, **E**, **F**, **H**, **I** = 10 µm.

Briefly, for all antibodies, slides were first deparaffinized, washed in phosphate buffer saline (PBS; pH 7.4) (3 × 5 minutes) and incubated in 0.3% H_2_O_2_ in PBS for 30 minutes to block endogenous peroxidase activity. Sections were then washed in PBS (3 × 5 minutes) and heat-induced antigen retrieval was performed in citrate buffer (pH 6.0) using an autoclave (121 °C for 5 minutes) and then cooled to room temperature for another 20 minutes. After washing with PBS, sections were incubated with primary antibodies overnight at room temperature (DCX: 1:1000; STMN1 1:80.000; Ki-67 1:2500; GFAP: 1:2500; Iba1: 1:1000). After another wash, sections were incubated with HRP-labelled Envision (K5007; DAKO, Glostrup, Denmark). Immunostaining was visualized with chromogen 3,3’-diaminobenzidine (DAB; K5007; DAKO). Finally, sections were counterstained with hematoxylin, dehydrated, and coverslipped with Quick D (Klinipath, Duiven, The Netherlands). Negative controls for each primary antibody were included by omitting the primary antibody and all showed no immunoreactivity. Per antibody, sections from all brain donors were included in one staining session in order to minimize variability between groups. Examples of cells positive for either DCX, STMN1 or Ki-67 immunoreactive signal are shown in Fig. 1. Furthermore, for images of the DCX stain of the complete granule layer see Supplementary Fig. 1.

### Quantification

The immunoreactive signals for DCX, GFAP and Iba1 were studied in the GCL, SGZ, and in the cornu ammonis 4 (CA4) / hilar region of the hippocampus. For quantification, images of the DAB-stained sections were collected using an Olympus BX4q microscope with a Leica MC 170HD digital camera with a 10x objective. Two photographs of the immunostainings of the DG (2592 ×1944 pixels; 10x) per slide were imported in ImageJ, and the surface area of the immunocytochemical detected threshold of the cellular signal occupied by immunoreactive signal, i.e. the DAB deposit, was quantified by means of ImageJ software (https://imagej.nih.gov/ij/) using the plug-in color deconvolution, as follows. The surface areas of the DG subregions were delineated in the section using the Haematoxylin counterstain, quantified, and expressed in µm2. Pixels with a value within the established threshold were then included in the percentage of positive area occupied by DAB (%AO) relative to the surface area of the outlined DG. For the thresholds, a value that made a clear distinction between positive cells and the background was selected and subsequently validated by an experienced neuropathologist, which resulted in a threshold of 135 for DCX, 147 for GFAP, and 181 for Iba1 of the maximum threshold of 255. The results of the two photographs per slide were averaged. Also, in donors where both hippocampi were available, results from the two hemispheres were averaged.

For STMN1 and Ki-67, whole-slide images were taken from the entire hippocampal surface at 20x magnification using an Olympus VS200 slide scanner. The GCL and SGZ of the DG were delineated and then quantified to calculate %AO (for STMN1 and Ki-67) using QuPath software (version v0.3.2; https://qupath.github.io/) [66]. For the percentage of positive cells, ROIs were drawn on the DG and SGZ and quantified using the positive cell detection workflow. The positive cells were then manually quantified per ROI and divided over the total number of cells present. This process with QuPath was also executed on the DCX images to calculate the percentage of positive DCX cells relative to the total number of cells present.

### Statistical analysis

Data were analyzed using the software “Statistical Package for the Social Sciences” (IBM SPSS Statistics for Windows, version 27.0, 2020). The analyses were performed using an ANOVA F-test to compare differences of means among the HC, DC and ECT group. When a significant difference between means was observed, Tukey’s HSD multiple comparisons were used to pairwise compare each group. Linear regression analyses were then used to assess associations between the markers DCX, STMN1, and Ki-67 consecutively as dependent variables and remission status, the number of ECT sessions, and the time interval between the last ECT and death as independent variables. All linear regression analyses were corrected for age of death to account for a possible age effect. As remission status was unknown for 3 ECT patients, linear regression analyses were executed both with remission status as an independent variable including a total of 9 ECT patients and without remission status as an independent variable including a total of 12 ECT patients. The significance level was set at *p* < 0.05.

## Results

### Descriptive statistics and pathology

The mean age of death of the donors was 68.87 (SD = 16.02) years for the HC group, 66.90 (SD = 13.47) for the DC group, and 54.25 (SD = 21.85) for the ECT group. There was no significant difference in age at death between the groups (F(2, 34) = 2.55, *p* = 0.093). Also, no significant difference was found in post-mortem delay (PMD) between the groups (F(2,34) = 1.12, *p* = 0.337), with a mean PMD of 7.30 hours (SD = 2.69) in the HC group, 6.13 hours (SD = 1.39) in the DC group and 7.65 hours (SD = 2.80) in the ECT group. In the HC group 46.7% was male, which was 40.0% for the DC group and 58.3% for the ECT group.

**Fig. 2:**
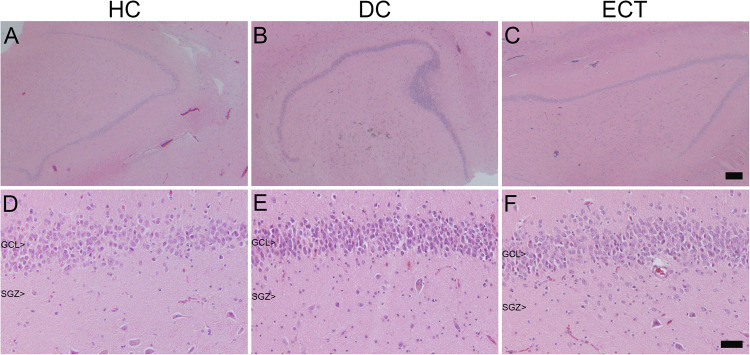
Representative photomicrographs of H&E stained tissue sections of the hippocampal dentate gyrus. The photomicrographs show the granule cell layer (GCL) and subgranular zone (SGZ) of; a healthy control donor (HC; **A** & **D**), a control donor with a depressive disorder (DC; **B** & **E**), and an ECT donor (ECT; **C** & **F**). Scale bar **A**–**C** = 250 µm, **D**–**F** = 50 µm.

Importantly, a histopathological study by our NBB neuropathologist found no indications for major cell loss, nor for gross cytoarchitectural malformations in any of the cell layers of the main hippocampal DG and CA subregions of the ECT, DC, or HC group (Fig. 2). The thickness of the GCL, as determined by averaging two measurements per case, was not significantly different between the groups (F(2, 34) = 0.33, *p* = 0.724). The cytoarchitecture of all hippocampal subareas appeared normal. There was a presence of mild age-related changes in neurofibrillary tangles or amyloid deposits in the hippocampus of most donors, which were matched between groups (Supplementary Table 1).

### Proliferation and neuronal maturation

**Fig. 3:**
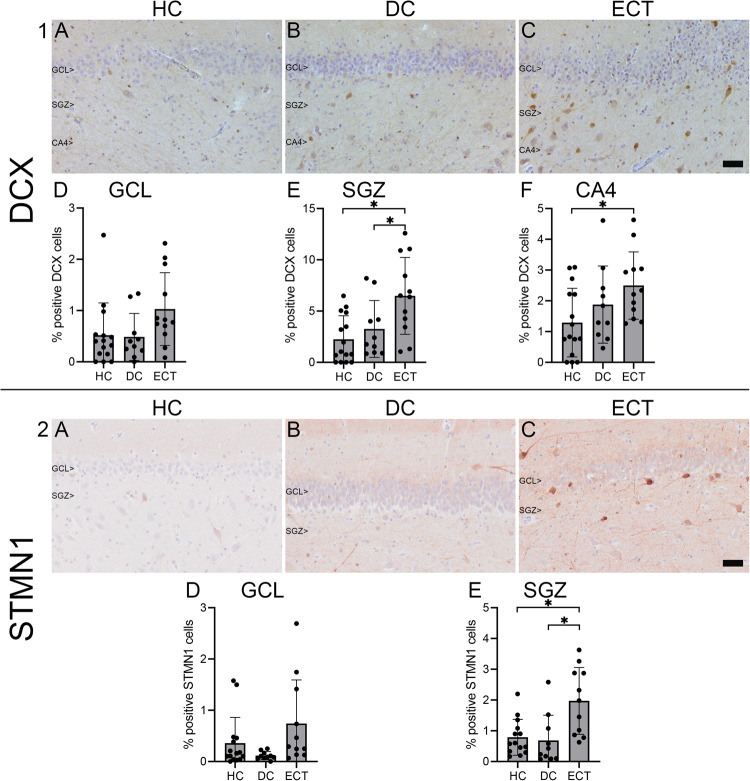
Doublecortin (DCX) and Stathmin 1 (STMN1) expression in the dentate gyrus. The first part shows representative images of DCX expression in the granule cell layer (GCL), subgranular zone (SGZ), and cornu ammnois 4 (CA4) in a healthy control donor (HC; 1**A**), a control donor with a depressive disorder (DC; 1**B**), and a donor who received electroconvulsive therapy during life (ECT; 1**C**). Note the substantial signal in the GCL, SGZ, and CA4 where many positive cells were found. Quantification of the overall optical density and the distribution of the groups is shown in percentages of positive DCX cells in the GCL (1**D**), SGZ (1**E**), and CA4 (1**F**). In the SGZ, the mean percentage of positive DCX cells was significantly higher in the ECT group than in the HC (*p* = 0.003) and DC (*p* = 0.040) groups. In the CA4, the mean percentage of positive DCX cells was also significantly higher in the ECT group than in the HC group (*p* = 0.027). The second part shows representative images of STMN1 expression in the GCL and SGZ in a HC (2**A**), a DC (2**B**), and a ECT donor (2**C**). Quantification is shown in percentage of positive STMN1 cells in the GCL (2**D**) and SGZ (2**E**). In the SGZ, the percentage of positive STMN1 cells was significantly higher in the ECT group than in the HC (*p* = 0.004) and DC (*p* = 0.004) groups. Scale bar = 50 µm.

The differences between groups in the mean percentage of positive DCX cells in the GCL (mean HC = 0.52, mean DC = 0.49, mean ECT = 1.03) was not significant (F(2, 34) = 2.90, *p* = 0.069, η²=0.15). However, these differences between groups were significant for both the SGZ (F(2, 34) = 7.14, *p* = 0.003, η²=0.30) and CA4 (F(2, 34) = 3.68, *p* = 0.036, η²=0.18). Next, Tukey’s HDS post hoc pairwise comparisons showed that in the SGZ, the mean percentage of positive DCX cells was significantly higher in ECT donors (mean=6.48) than in both the HC (mean=2.25, *p* = 0.002) and DC (mean = 3.26, *p* = 0.040) groups (Fig. 3). Additionally, in CA4, the mean percentage of positive DCX cells of the ECT donors (mean = 2.50) was significantly different compared to the HC donors (mean = 1.29, *p* = 0.027), but not compared to the DC donors (mean=1.88, *p* = 0.429). Furthermore, no significant difference was found between the HC and DC groups in the SGZ (*p* = 0.683) or CA4 (*p* = 0.431). Regarding the mean %AO by DCX, the ANOVA showed significant differences between groups in the GCL (F(2, 34) = 3.73, *p* = 0.034, η²=0.18), SGZ (F(2, 34) = 5.76, *p* = 0.007, η²=0.25), and CA4 (F(2, 34) = 4.90, *p* = 0.014, η²=0.22). Next, pairwise comparisons showed that in the GCL, the mean %AO by DCX was significantly different between the healthy control donors and ECT donors (mean HC = 0.09, mean ECT = 0.39, *p* = 0.037). Moreover, in the GCL, no significant differences were found in the mean %AO by DCX between the DC group (mean=0.12) and the HC group (*p* = 0.959), or the ECT group (*p* = 0.111). In the SGZ, a significantly higher %AO by DCX was also observed in ECT donors (mean ECT = 1.02) compared to healthy control donors (mean HC = 0.16, *p* = 0.007). The difference in %AO by DCX between the ECT group and the DC group (mean=0.31) was close to significance (*p* = 0.051). No significant difference in %AO by DCX in the SGZ was found between both control groups (*p* = 0.855). Finally, in CA4, the mean %AO by DCX was also significantly higher in ECT donors (mean ECT = 0.59) compared to the HC group (mean HC = 0.07, *p* = 0.011). Furthermore, no significant differences were found in CA4 in the mean %AO by DCX between the DC group (mean = 0.22) and the HC group (*p* = 0.696), or the ECT group (*p* = 0.125). In order to investigate the potential impact of age, ANCOVA’s were executed with age as covariate, which showed no significant effect of age on the DCX results. Therefore, the results of the ANOVA were displayed in order to also assess the Tukey’s HSD post-hoc pairwise comparisons between the groups.

For the analyses of STMN1, data of one ECT donor had to be excluded due to poor tissue quality. No significant differences were found between groups in the mean %AO by STMN1 in both the GCL (mean HC = 1.84, mean DC = 0.30, mean ECT = 3.67; F(2, 33) = 1.45, *p* = 0.248, η²=0.08) and SGZ (mean HC = 1.09, mean DC = 0.23, mean ECT = 1.51; F(2, 32) = 1.78, *p* = 0.186, η²=0.10). However, the mean percentage of positive STMN1 cells was significantly different between the groups in the SGZ F(2, 32) = 8.15, *p* = 0.001, η²=0.34. Tukey’s HSD post-hoc pairwise comparisons showed that in the SGZ, the mean percentage of positive STMN1 cells was significantly increased in the ECT group (mean = 1.97) compared to both the HC group (mean = 0.79; *p* = 0.004) and the DC group (mean = 0.69; *p* = 0.004; Fig. 3). This mean percentage was not significantly different between the HC and DC group (*p* = 0.953). In the GCL, the mean percentage of positive STMN1 cells was also higher in the ECT group (mean=0.74) than in the HC (mean = 0.36) and DC (mean = 0.12) group, which was close to significance F(2, 33) = 3.23, *p* = 0.052, η²=0.16).

**Fig. 4:**
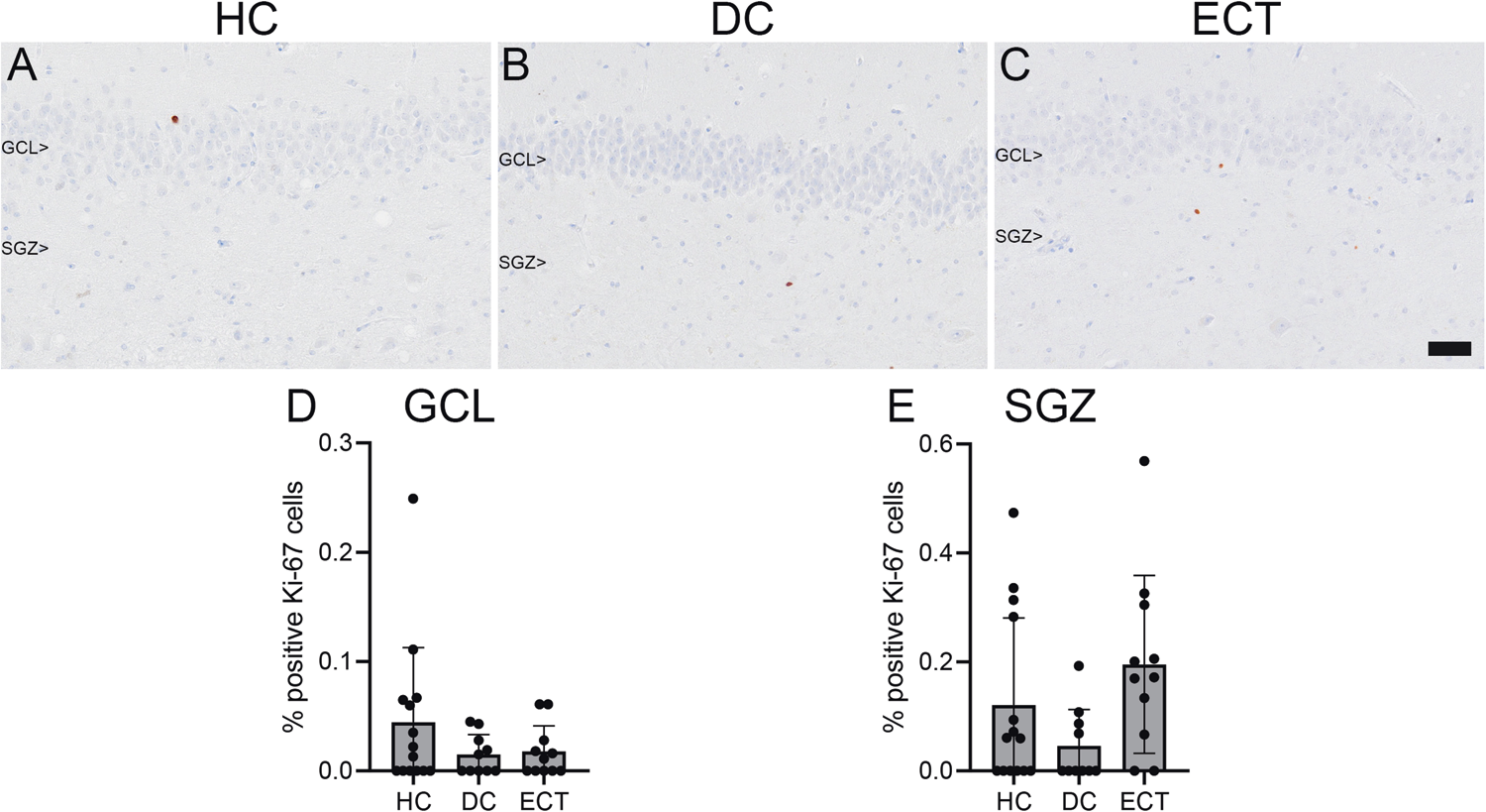
Ki-67 expression in the granule cell layer (GCL) and subgranular zone (SGZ). Representative examples of Ki-67 positive cells in a healthy control donor (HC; **A**), a control donor with a depressive disorder (DC; **B**), and a donor who received electroconvulsive therapy during life (ECT; **C**). There were no significant differences between the three groups in percentages of positive Ki-67 cells in the GCL (**D**) and SGZ (**E**). Scale bar = 50 µm.

For the analyses of the proliferation marker Ki-67, data of one ECT donor and one healthy control donor had to be excluded due to poor tissue quality. After exclusion, the percentage of positive Ki-67 cells was not significantly different between the groups in both the GCL (mean HC = 0.044, mean DC = 0.015, mean ECT = 0.018; F(2, 32) = 1.53, *p* = 0.233, η²=0.09) and SGZ (mean HC = 0.121, mean DC = 0.046, mean ECT = 0.195; F(2, 32) = 2.94, *p* = 0.067, η²=0.16), indicating no significant differences between the three groups in cell proliferation (Fig. 4). Furthermore, no significant differences were found between groups in the mean %AO for the number of cells that were immune-positive for the proliferation marker Ki-67 in both the GCL (mean HC = 0.023, mean DC = 0.003, mean ECT = 0.010; F(2, 32) = 1.53, *p* = 0.233, η²=0.09) and SGZ (mean HC = 0.029, mean DC = 0.005, mean ECT = 0.011; F(2, 32) = 1.62, *p* = 0.213, η²=0.09).

Within the ECT group, none of the independent variables (remission status, number of ECT sessions, and time interval between the last ECT and death of the patient) were significantly associated with a percentage of positive DCX, STMN1, or Ki-67 cells in either the GCL or SGZ (Supplementary Table 2).

### Inflammation and immune response

**Fig. 5:**
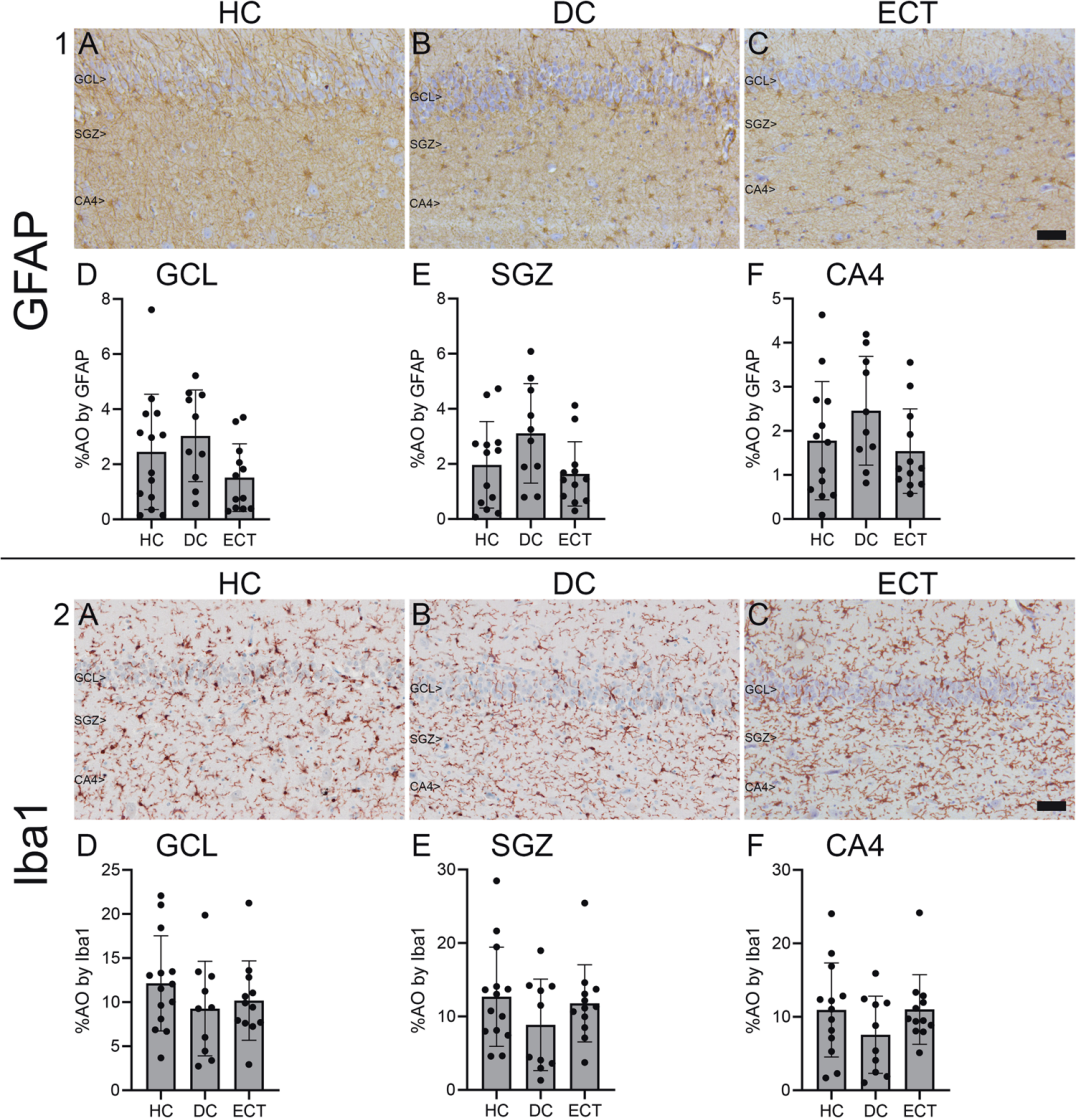
Glial fibrillary acidic protein (GFAP) and Ionized calcium binding adaptor molecule 1 (Iba1) expression in the dentate gyrus. The photomicrographs show the expression in the granule cell layer (GCL), subgranular zone (SGZ) and cornu ammonis 4 (CA4) in a healthy control donor (HC; **A**), a control donor with a depressive disorder (DC; **B**), and a donor who received electroconvulsive therapy during life (ECT; **C**). The distribution of the groups is shown in percentages of cell area occupied (%AO) by GFAP or Iba1 in the GCL (**D**), SGZ (**E**), and CA4 (**F**). No differences in mean %AO by GFAP or Iba1 were found between the groups. Scale bar=50 µm.

For our analyses of inflammatory changes, data of one healthy control donor had to be excluded due to poor tissue quality. No differences were found in %AO in the GCL (Iba1: F(2, 33) = 1.01, *p* = 0.375, η²=0.06; GFAP: F(2, 33) = 2.04, *p* = 0.147, η²=0.11), SGZ (Iba1: F(2, 33) = 1.18, *p* = 0.321, η²=0.07; GFAP: F(2, 32) = 2.81, *p* = 0.075, η²=0.15) or CA4 (Iba1: F(2, 32) = 1.36, *p* = 0.272, η²=0.08, GFAP: F(2, 32) = 1.96, *p* = 0.157, η²=0.11) between donor groups (Fig. 5), indicating no signs for a strong inflammatory activation as a result of ECT, as reflected by conventional microglia and astrocyte markers.

## Discussion

For the first time, cytoarchitectural, neuropathological, glia- and neuroplasticity-related changes were explored in the human post-mortem hippocampus in a unique group depressed donors treated with ECT (ECT), compared with depressed donors who had not received ECT (DC) and neurologically healthy control donors (HC). We found no apparent structural damage of the ECT treatment in the hippocampus. While the numbers of proliferating cells did not differ between the groups, we found DCX expression to be significantly higher in the DG of ECT patients relative to neurologically healthy control donors. Moreover, in the SGZ, where most proliferation and differentiation is expected to occur [22, 23, 25, 26], the ECT donors even had a significantly higher percentage of cells expressing DCX compared to the depressed controls. Furthermore, the percentage of cells expressing STMN1 in the SGZ was significantly higher in ECT donors compared to both healthy control donors and depressed controls.

### Neuropathology

Importantly, we found no evidence for overt neuropathology, neuroinflammation or changes in cytoarchitecture in the hippocampi of ECT-treated donors. This is in line with three case reports showing neither neuronal damage nor significant abnormalities in the brains of old donors aged 84-92, who received 91-1250 ECT sessions during their lives [5–7]. Similar age-related pathological changes were found in all groups, including hippocampal tau and amyloid pathology.

### Hippocampal neuroplasticity

The significant increase in the percentage of cells expressing STMN1 and DCX in ECT donors supports the concept that hippocampal neuroplasticity is involved in the ECT treatment [37, 67]. This increased expression in the ECT group indicates that also the human hippocampal DG of depressed patients retains considerable structural plasticity. We did find Ki-67 expression in the hippocampus in our donors, showing that neurogenesis occurs in the adult human brain [58, 63, 68–72]. Furthermore, we saw no differences in Ki-67 immunoreactivity and percentage of cells expressing Ki-67 between our groups, which may indicate that neurogenesis was not increased as a result of ECT in our sample. However, this could also be due to the low frequency of this particular proliferation marker in thin sections of human hippocampi of subjects this age, which is also reported in previous studies [58, 63, 68–72]. Another explanation could be that as the expression of Ki-67 is short-lived [73], we were not able to pick up possible differences induced by ECT. Our donors had varying and rather long delays between their ECT sessions and time of death, and neurogenesis could thus have taken place, but is likely not reflected by Ki-67 signal in these tissues. An alternative explanation could be that as a potent stimulus, ECT might also have enabled existing neurons in the DG and SGZ to undergo ‘de-maturation’, i.e. in the absence of active new cell formation, via which route, mature cells could possibly become activated and rewire again, as proposed before [67]. Furthermore, the increased DCX expression in the ECT group is also present in the CA4, which aligns with some of the changes found after epileptic activity in rodent models, such as the migration of newborn neurons into the hilus [3], and also in humans [49].

The findings regarding DCX and the higher percentage of STMN1 positive cells in the SGZ in ECT donors may together indicate an increase in neuronal plasticity after ECT [61]. While both the STMN1 immunoreactivity and the percentage of cells expressing STMN1 was higher in the ECT donors, this difference was not significant for the STMN1 immunoreactivity. This could be due to various reasons, such as cell density and clustering, differences in staining intensity, cell morphology and their distribution. Furthermore, due to the small sample sizes in each group, statistical significance may not be reached, even if there are substantial differences. Due to the nature of these marker proteins and the neurogenic stages they reflect, the current data suggests that in the SGZ more cells may be transitioning from a neuronal precursor to a young neuronal stage after ECT. Whether these cells will actually all develop into functional DG neurons and whether it is indeed adult neurogenesis that contributes to the structural and functional changes seen after ECT in depression [36, 74], awaits further studies.

In the present study, characteristics such as remission status after ECT, the total number of ECT sessions and the interval between the last ECT and death of the patient were not correlated with the expression of the markers studied. This could be due to various reasons including our current sample size and variation between the patients in bilateral vs unilateral placement, and to differences in subsequent ECT (maintenance) treatment regimes. Future studies with a larger sample size and more homogenous and/or better-stratified ECT patient groups are needed to explore such relations in more detail.

Although DCX expression was higher in depressed donors with ECT treatment than in depressed donors without ECT treatment, this difference was only significant in the SGZ. It is likely that the use of antidepressants in both groups influenced DCX, since antidepressants can stimulate neuroplasticity, as reported before mostly in young individuals [14–17, 63, 75]. However, we found a significantly higher expression of DCX in the DG of the ECT group than in the HC group, while no difference in DCX results were found between the HC and DC group, indicating the effect of ECT rather than antidepressants. Additionally, the presence of STMN1-positive cells in the SGZ was significantly higher in the ECT group compared to both the HC and DC groups. To truly understand the contribution of medication on DCX expression, an additional group of non-medicated depressed controls is needed which is not available.

### Neuroinflammation

No significant differences in the expression of immune-related or inflammatory markers were found between ECT and control donors in the present study. A systematic review showed that an acute and peripheral immuno-inflammatory response is present immediately after an ECT session, while over the long term, at the end of the ECT course, this inflammatory response is absent and may even be reversed [53]. Therefore, our results demonstrate that increased hippocampal volume following ECT is possibly related to effects on neuroplasticity and is less likely due to inflammation.

### Limitations and considerations

In this archival human post-mortem study, the mid-level region of the hippocampus was unavailable in 21.6% of cases since this NBB tissue is also used by other research groups. The tissue that was used instead in those cases was however adjacent to the midlevel region and hence quite comparable. We therefore do not expect that anatomical differences will have contributed much to our results. Overall, we expect effects of a general stimulus like ECT on neuroplasticity to be similar from the anterior through the posterior part of the hippocampus.

The types of antidepressant drugs taken by the patients in our current DC and ECT groups, differed slightly; the serotonin-noradrenaline reuptake inhibitors and monoamine oxidase inhibitors had only been taken by the ECT group. While some exceptions exist [18], rodent studies have generally shown comparable effects of different types of antidepressant medication; when prescribed for a sufficiently long period of time, they almost all stimulate hippocampal proliferation and/or neurogenesis, parallel to a suppression of the depressive and/or anxiety-like behaviors [11, 13]. Furthermore, the ECT and DC groups were comparable in terms of the total number of life-time depressive episodes (ECT: mean = 3.38, (*n* = 8); DC: mean = 4.11 (*n* = 9); *p* = 0.477) and age at first depressive episode (ECT: mean = 31.55, (*n* = 11); DC: mean = 34.67, (*n* = 9), *p* = 0.614), showing no significant differences between the groups using ANOVA. Nonetheless, it remains possible that during an episode, the depression in the ECT group was more severe, which is hard to compare with our current data and hence remains a limitation of this study.

Although age is likely to be a contributing factor to differences in neurogenesis [23, 24, 63], the average ages in our cohort were not significantly different between the groups. Neurogenesis had been reported to gradually decrease with advancing age, but the extent of neurogenesis that is expected to be present between the mean ages of 54 and 69, i.e. of the patients that were studied here, probably did not change substantially between these ages and is likely quite comparable [23]. As such, age is unlikely to have contributed much to our current results.

Technical aspects can also influence the expression of neuroplasticity markers. In comparison to Ki-67 that is present in proliferating cells for a short period of time with a half-life of approximately 90 minutes [73], STMN1 has a broader role in cellular processes and its presence and detection may not be as tightly linked to specific time frames [60, 61]. DCX is a maturation marker that is also present for a longer period of time; i.e. between 2 and 14 days after birth of a newly generated cell, at least in rodents [46, 48] and a DCX signal thus has a higher chance of being detected in thin tissue sections than Ki-67. The expression of DCX is initiated at varying times after cell cycle exit with variable timeframes, but with an average duration of approximately three weeks [46–48, 76]. Although other studies have carefully compared tissue conditions and have reported that DCX can be optimally detected in post-mortem tissue with a short fixation of 24 hours [23, 77], we and others have used optimized protocols and could still detect DCX signal in human brains fixed for a longer period [27, 49, 50, 78].

The differences between studies might thus be due to (combinations of) different epitopes of DCX, the use of different antibodies, differences in tissue handling and fixation, and also to variations in post-mortem delay. Although no such correlation was found in a human study with short fixation times [77], an increase in PMD reduced DCX immunoreactive signal, especially in older post-mortem rat brains [58, 77]. Importantly, the mean PMD in our current sample is 7.10 hours (SD = 2.50) which is relatively short compared to other human post-mortem studies and can thus be seen as a major advantage with respect to tissue and antigen preservation, especially when compared to studies with much longer PMDs [19, 57].

## Conclusions

We reported for the first time an upregulation of the percentage of cells expressing STMN1 and DCX, but not of proliferation, in the DG of donors who had received ECT during their lives, notably in the absence of any indications for major hippocampal injury, for classic neuropathology or neuroinflammation. These first, explorative results on the human hippocampus support the involvement of neuroplasticity in the antidepressant effects of ECT and are in line with earlier rodent and human MRI studies. As such, they provide new insights into the role of brain plasticity in depression and in the antidepressant action of ECT.

### Supplementary information

Supplemental information

Supplementary Figure 1
