# Supplementary material for: Electroconvulsive therapy is associated with increased immunoreactivity of neuroplasticity markers in the hippocampus of depressed patients
Source: Transl Psychiatry. 2023 Nov 20;13:355. doi: 10.1038/s41398-023-02658-1 (PMC10658169; doi:10.1038/s41398-023-02658-1)
Supplement: Supplementary file 1 — Supplemental information [file 41398_2023_2658_MOESM1_ESM.docx]

**Supplemental information**

**Supplementary Table 1.** Neuropathological characteristics of all included donors.

| Case | Group | Gender | Age of death | Cause of death | PMD | Braak | Amyloid | pH liquor |
| --- | --- | --- | --- | --- | --- | --- | --- | --- |
| 1 | ECT | Male | 55 | Suicide | >12:00 | 1 | n/a | n/a |
| 2 | ECT | Male | 73 | Cachexia / dehydration | 5:15 | 2 | B | 6.38 |
| 3 | ECT | Female | 100 | Natural death | 5:50 | 2 | B | 6.40 |
| 4 | ECT | Female | 47 | Legal euthanasia due to severe MDD | 6:05 | 0 | O | n/a |
| 5 | ECT | Male | 60 | Cachexia | 5:00 | n/a | n/a | 6.20 |
| 6 | ECT | Male | 58 | Legal euthanasia due to chronic MDD | 4:30 | 1 | n/a | 6.80 |
| 7 | ECT | Female | 45 | Legal euthanasia due to multiple psychiatric disorders | 7:30 | 1 | O | 6.93 |
| 8 | ECT | Female | 46 | Legal euthanasia due to treatment-resistant bipolar disorder | 5:45 | 1 | A | n/a |
| 9 | ECT | Female | 23 | Legal euthanasia due to multiple psychiatric disorders | 8:35 | 0 | O | n/a |
| 10 | ECT | Male | 48 | Legal euthanasia due to refractory MDD | 12:45 | 1 | A | 6.76 |
| 11 | ECT | Male | 75 | Legal euthanasia due to progressive MS | 9:05 | n/a | n/a | n/a |
| 12 | ECT | Male | 21 | Legal euthanasia due to multiple psychiatric disorders | 8:55 | 0 | O | 6.73 |
| 13 | DC | Female | 75 | Acute abdomen | 4:00 | 1 | n/a | n/a |
| 14 | DC | Female | 62 | Cachexia by gall bladder carcinoma | 5:00 | 1 | O | 6.11 |
| 15 | DC | Male | 72 | Dehydration and cachexia. Lithium intoxication. | 4:35 | 1 | A | 6.40 |
| 16 | DC | Female | 66 | Legal euthanasia due to multiple psychiatric disorders | 7:55 | 1 | O | n/a |
| 17 | DC | Male | 64 | Infection, dehydration, cachexia | 8:05 | 0 | O | 6.37 |
| 18 | DC | Female | 58 | Renal insufficiency, termination dialysis | 7:20 | 0 | O | 5.61 |
| 19 | DC | Male | 38 | Legal euthanasia due to physical and mental impairments | 6:15 | 0 | O | 6.79 |
| 20 | DC | Female | 70 | Terminal kidney failure | 6:00 | 2 | n/a | 6.95 |
| 21 | DC | Male | 74 | Respiratory insufficiency, metastatic prostate cancer | 5:30 | 4 | n/a | 6.18 |
| 22 | DC | Female | 90 | Myocardial infarction | 6:40 | 2 | n/a | 6.51 |
| 23 | HC | Female | 61 | Legal euthanasia due to ovary carcinoma | 6:50 | 0 | O | 6.50 |
| 24 | HC | Female | 73 | Respiratory failure after acute subdural hematoma with cerebral entrapment | 6:40 | 1 | A | n/a |
| 25 | HC | Female | 50 | Metastasized large cell bronchocarcinoma | 4:10 | 1 | O | 6.98 |
| 26 | HC | Female | 84 | Myelodysplasia | 6:55 | 1 | O | n/a |
| 27 | HC | Female | 60 | Infection from unknown causes | 7:30 | 1 | A | 6.8 |
| 28 | HC | Male | 51 | Suicide by refusing food and water | 7:45 | 0 | O | 7.05 |
| 29 | HC | Male | 55 | Legal euthanasia due to esophageal cancer | 7:30 | 0 | O | 6.88 |
| 30 | HC | Male | 102 | Ileus | 5:00 | 3 | A | 6.64 |
| 31 | HC | Male | 96 | Urinary tract infection | 4:10 | 4 | B | 6.09 |
| 32 | HC | Female | 60 | Legal euthanasia due to metastatic mammary carcinoma | 5:30 | 0 | O | 7.07 |
| 33 | HC | Female | 68 | Metastatic pancreatic cancer | 3:30 | 0 | n/a | 7.37 |
| 34 | HC | Male | 68 | Myocardial infarction | <12 | 0 | O | n/a |
| 35 | HC | Female | 70 | Myocardial infarction | <10 | 3 | A | n/a |
| 36 | HC | Male | 53 | Dissection aorta | <12 | 3 | A | n/a |
| 37 | HC | Male | 82 | Myocardial infarction | <12 | 3 | B | n/a |

The Braak stage is a score for Alzheimer’s disease pathology referring to tau pathology (1) (0=no pathology up to 6=severe pathology), amyloid stands for amyloid pathology (O=no amyloid deposits; A, B, and C represent increasing amyloid deposits). Abbreviations: PMD=post-mortem delay (in hours); ECT=donors who received electroconvulsive therapy; DC=control donors with an established depressive disorder; HC=neurologically and psychiatrically healthy control donors; n/a=not available.

**Supplementary Table 2.** Linear regression analyses of the association between ECT-related characteristics and expression of doublecortin (DCX), Stathmin 1 (STMN1), and Ki-67.

|  | DCX | | STMN1 | | Ki-67 | |
| --- | --- | --- | --- | --- | --- | --- |
|  | GCL | SGZ | GCL | SGZ | GCL | SGZ |
| Remission status | β=0.31, SE=0.37, p=0.442 | β=0.18, SE=1.60, p=0.914 | β=-0.49, SE=0.48, p=0.377 | β=-0.05, SE=0.37, p=0.897 | β=-0.004, SE=0.01, p=0.765 | β=-0.07, SE=0.04, p=0.141 |
| Number of ECT sessions | β=0.002, SE=0.01, p=0.881 | β=0.01, SE=0.06, p=0.927 | β=-0.01, SE=0.02, p=0.635 | β=0.001, SE=0.01, p=0.937 | Β<-0.001, SE<0.001, p=0.407 | Β=0.001, SE=0.001, p=0.515 |
| Time interval between last ECT and death | β=-0.02, SE=0.03, p=0.551 | β=-0.06, SE=0.12, p=0.657 | β=-0.01, SE=0.03, p=0.718 | β=0.01, SE=0.02, p=0.640 | Β<-0.001, SE=0.001, p=0.723 | β=-0.01, SE=0.002, p=0.091 |

Only the results of the linear regression analyses with remission status as independent variable are displayed, since the results without remission status were similar. Abbreviations: ECT=electroconvulsive therapy; GCL=granule cell layer; SGZ=subgranular zone.

**Supplementary Figure 1.**

DCX immunoreactivity throughout the granule layer in a healthy control donor (HC; A), a control donor with a depressive disorder (DC; B), and a donor who received electroconvulsive therapy during life (ECT; C). Scale bar = 500 µm.

**References**

1. Braak H, Braak E. Neuropathological stageing of Alzheimer-related changes. Acta Neuropathol. 1991;82(4):239-59.
