# Supplementary figures and images for: Electroconvulsive therapy is associated with increased immunoreactivity of neuroplasticity markers in the hippocampus of depressed patients
Source: Transl Psychiatry. 2023 Nov 20;13:355. doi: 10.1038/s41398-023-02658-1 (PMC10658169; doi:10.1038/s41398-023-02658-1)

# Doublecortin (DCX)

Control

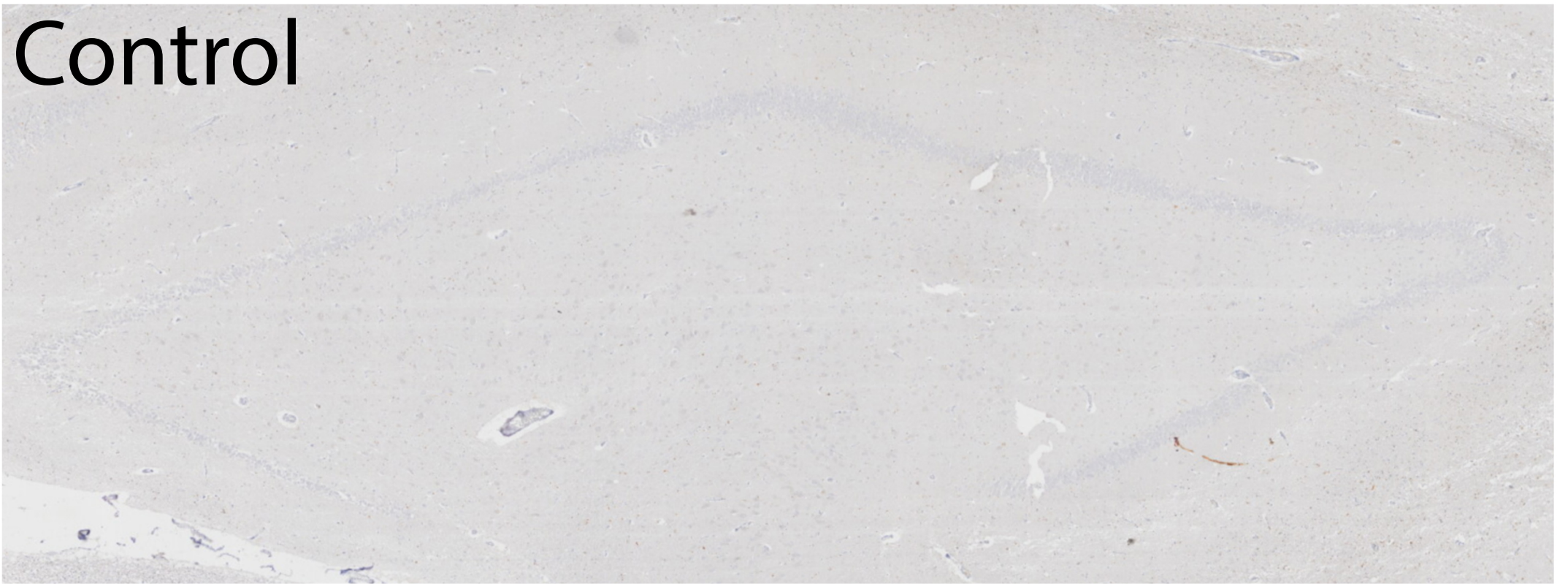

DC

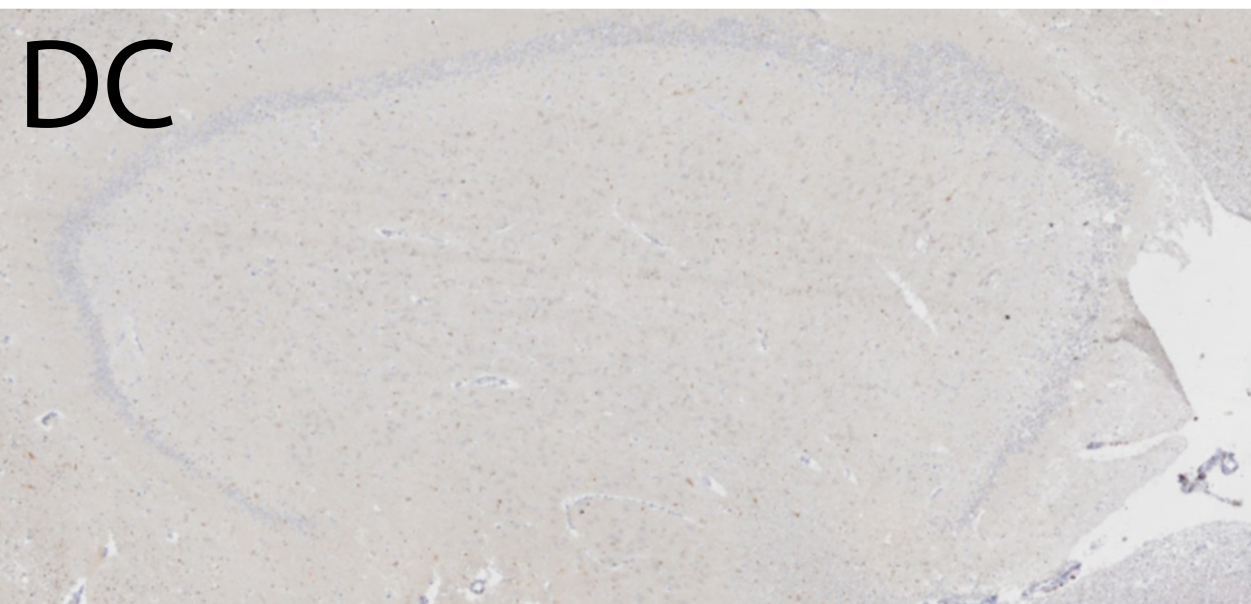

ECT

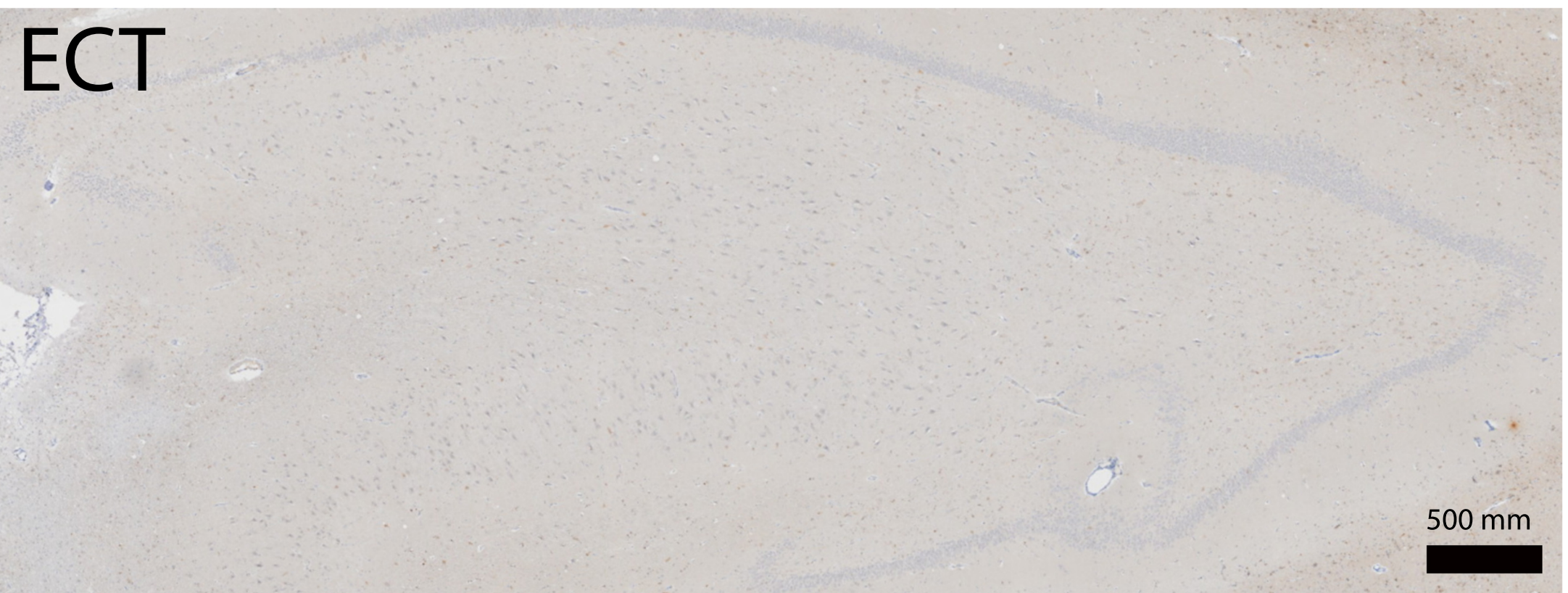

Supplement: Supplementary file 2 — Supplementary Figure 1 [file 41398_2023_2658_MOESM2_ESM.pdf]
